# Exceptional Properties of *Lepidium sativum* L. Extract and Its Impact on Cell Viability, Ros Production, Steroidogenesis, and Intracellular Communication in Mice Leydig Cells In Vitro

**DOI:** 10.3390/molecules27165127

**Published:** 2022-08-11

**Authors:** Tomas Jambor, Terezia Zajickova, Julius Arvay, Eva Ivanisova, Ivana Tirdilova, Nikola Knizatova, Hana Greifova, Anton Kovacik, Eliska Galova, Norbert Lukac

**Affiliations:** 1 Institute of Applied Biology, Faculty of Biotechnology and Food Sciences, Slovak University of Agriculture in Nitra, Tr. A. Hlinku 2, 949 76 Nitra, Slovakia; 2 Department of Genetics, Faculty of Natural Sciences, Comenius University in Bratislava, Ilkovicova 6, 842 15 Bratislava, Slovakia; 3 Institute of Food Science, Faculty of Biotechnology and Food Sciences, Slovak University of Agriculture in Nitra, Tr. A. Hlinku 2, 949 76 Nitra, Slovakia; 4 AgroBioTech Research Centre, Department of Food Technology, Slovak University of Agriculture, Tr. A. Hlinku 2, 949 76 Nitra, Slovakia

**Keywords:** Leydig cells, *Lepidium sativum* L., viability, reactive oxygen species, steroidogenesis, intracellular communication

## Abstract

The prevalence of reproductive dysfunction in males has risen in the last few years, and alternative therapies are gradually gaining in popularity. Our in vitro study aimed to evaluate the potential impact of *Lepidium sativum* L. on mice TM3 Leydig cells, concerning basal parameters such as cell viability, cell membrane integrity, and lysosomal activity, after 24 h and 48 h exposure. Moreover, reactive oxygens species generation, sex-steroid hormone secretion, and intercellular communication were quantified. In the present study, the microgreen extract from Lepidium was rich in ferulic acid, 4-OH benzoic acid, and resveratrol, with a significant antioxidant activity. The results showed that lower experimental doses (62.5–250 µg/mL) could positively affect the observed parameters, with significant differences at 250 µg/mL after 24 h and 48 h, respectively. Potential risks could be associated with higher concentrations, starting at 500 µg/mL, 1000 µg/mL, and 2000 µg/mL of *Lepidium.* Nevertheless, biochemical quantification indicated a significant antioxidant potential and a rich content of biologically active molecules at the applied doses, and time determined the intracellular response of the cultured model.

## 1. Introduction

Today, the increasing prevalence of subfertility or infertility has become a global problem. Therefore, the development of new therapeutic approaches and drugs, as an alternative to conventional therapy for infertility, is taking place across the medical and scientific fields [1]. The fertility rate of a couple can be influenced by several factors, including the age of each partner [2,3], and exposure to different environmental toxins [4], drugs [5], or radiation [6]. The presence of severe systemic diseases [7,8], specific disorders such as hormonal dysfunction [9] and varicocele [10], or an unhealthy lifestyle [11] also play crucial roles in this issue. Lower sexual desire, erectile dysfunction, abnormal sperm morphology, decreased motility, and hormonal imbalance are accompanying symptoms of developing infertility [12]. In general, male factor fertility issues are typically less problematic in terms of diagnostics and treatment, and preventive care might contribute to the improvement of fertility for some men as well. Besides the traditional therapy for male infertility (hormonal treatment or surgical intervention), natural compounds with stimulatory effects on male reproductive health may also provide an option for prevention and/or treatment [13]. In recent years, the herb *Lepidium sativum* L. has come to the forefront of infertility research. This edible plant naturally occurs in different regions of the world and belongs to the family Brassicaceae. Besides this, *Lepidium sativum* L. is a rich source of amino acids such as glutamic and aspartic acids, as well as fatty acids, including stearic and palmitic acid, which affect the nutritional and therapeutic potential in various diseases treatment. In addition, the unique potential of *Lepidium* was confirmed by different cellular models in vitro and supported by conclusions from rodent and human studies in vivo [14,15]. The benefits of *Lepidium* regarding the reproductive functions in males confirmed that using *Lepidium* extract might stimulate spermatogenesis, via the improvement of sperm mobility and total sperm concentration. Moreover, elongation of spermatozoa heads correlated with their improved fluidodynamic movement and improvement of sperm functionality [15,16]. Effects of *Lepidium* were also studied in terms of in vitro fertilization using mice and human cells [17]. The addition of *Lepidium* extract to the culture medium led to an increased fertilization rate, by improving the acrosome reaction of sperm cells and their motility toward an egg cell. In addition, *Lepidium* could affect steroidogenesis and enhance the production of sex-steroid hormones. An increased steroidogenic activity of Leydig cells was recorded [18]. Although the exceptional properties, such as antioxidant capacity [19], anti-inflammatory activity [20], anticancer potential [21], the hepatoprotective effect [22], and antibacterial strong [23], of *Lepidium* are well known, the exact molecular mechanism of action has not been fully elucidated.

Even though herbal extracts are currently commercially available in the form of various nutritional supplements, the exact mechanism of action of the individual constituents has been insufficiently characterized. Additionally, disruptions of cell morphology, inhibition of inter-and intracellular communication, damage to signaling pathways, affected redox status, and other changes in the cell microenvironment have been characterized to a limited extent. Therefore, our study aimed to identify the effect of *Lepidium sativum* L. on the components of the male reproductive system at the cellular level, using an in vitro system, TM3 Leydig cells. The characterization of *Lepidium’s* properties and identification of the cellular effects, including viability, membrane integrity, lysosomal activity, reactive oxygens species (ROS) production, intracellular communication, and sex-steroid hormone secretion, could reveal advantages for experimental extract applications.

## 2. Material and Methods

### 2.1. Plant Material Collection and Processing

*Lepidium sativum* L. was cultivated as a microgreen and purchased from the local producer (Microgreens Slovakia s.r.o., Bratislava, Slovakia). *Lepidium* was germinated (for 3 days) and cultivated (3–4 days) in 4 trays (dimension 5600 mm × 2800 mm) in a cultivation chamber under a 16/8 h day/night regime at 22–24 °C, with a relative air humidity of 40–50%. Fresh plant material was dried at room temperature, mechanically comminuted, and weighed in a quantity corresponding with the numbers of performed analyses. For the quantification of the total polyphenols, 1 g of the crushed *Lepidium sativum* L. was extracted with 10 mL 80% (*v*/*v*) ethanol (EtOH; CentralChem, Bratislava, Slovakia) for 8 h, with constant shaking at room temperature. After centrifugation (9000 rpm, 5 min), the supernatant was collected, filtered through a PVDF syringe filter (0.45 µm), and used for further experiments. In addition, the same procedure was used for the assessment of the total antioxidant capacity of the prepared extract [24].

For the high-performance liquid chromatography (HPLC) analysis, 2 g of dried *Lepidium sativum* L. was extracted by adding 20 mL of 80% (*v*/*v*) aqueous methanol (HPLC grade, Sigma-Aldrich, St. Louis, MO, USA). The mixture was shaken on a horizontal shaker for 24 h at room temperature. Afterward, the microgreen extract was filtered through the Whatman filter paper (Whatman, Maidstone, UK) and kept in a fridge (4 °C) until HPLC analyses [25]. 

The preparation for the in vitro experiments was as follows: Microgreens of *Lepidium sativum* L. were carefully harvested, immediately dried at room temperature, milled, weighed, and extracted in 80% (*v*/*v*) ethanol for 24 h in the dark. To remove any residual ethanol, the crude extract was subjected to evaporation (Stuart RE300DB rotary evaporator, Bibby Scientific Limited, UK) under reduced pressure (vacuum pump KNF N838.1.2KT.45.18, Freiburg, Germany) at 40 °C. Finally, the crude extract was dissolved in a dimethyl sulfoxide (DMSO; Sigma-Aldrich, St. Louis, MO, USA) and adjusted to 2000 µg/mL, which served as a stock solution [26,27].

### 2.2. Biochemical Analysis of the Extract

The total polyphenols quantity of the experimental extract was determined by the method used in a previous study [28], with slight modifications. A quantity of 100 µL of *Lepidium sativum* L. was mixed with the same amount of the Folin-Ciocalteu’s phenol reagent (Sigma-Aldrich, St. Louis, MO, USA), 1000 µL 20% (*w*/*v*), Na2CO3 (sodium carbonate; CentralChem, Bratislava, Slovakia), and 8.8 mL of distilled water. Followed by 30 min of incubation in the dark. The absorbance was measured using a spectrophotometer Jenway 6405 UV/VIS (Fischer Scientific, Leicestershire, UK) at a 700-nm wavelength. The total polyphenol amounts were expressed in mg/g gallic acid equivalents (GAE) (gallic acid; Sigma-Aldrich, St. Louis, MO, USA) [29].

The total flavonoid amount of *Lepidium sativum* L. was measured with the method used in [30]. A quantity of 500 µL of the experimental extract was mixed with 100 µL of 10% (*w*/*v*) ethanolic solution of aluminum chloride (Sigma-Aldrich, St. Louis, MO, USA), 100 µL of 1M potassium acetate (CentralChem, Bratislava, Slovakia), and 4.3 mL distilled water, followed by 30 min of incubation in the dark. The absorbance was measured using a spectrophotometer, Jenway 6405 UV/VIS (Fischer Scientific, Leicestershire, UK), at a 415-nm wavelength. The total flavonoid quantities were expressed in mg/g quercetin equivalents (QE) (quercetin; Sigma-Aldrich, St. Louis, MO, USA) [31].

The total amount of phenolic acid of the experimental extract was measured with a method used previously [32]. A quantity of 500 µL of *Lepidium sativum* L. was mixed with 500 µL of 0.5 M hydrochloric acid (CentralChem, Bratislava, Slovakia), 500 µL of Arnova reagent (mixture of 10% Na_2_MoO_4_ and 10% NaNO_2_), 500 µL of 1 M sodium hydroxide (*w*/*v*) (CentralChem, Bratislava, Slovakia), and 500 µL of distilled water. Subsequently, the absorbance was measured using a spectrophotometer, Jenway 6405 UV/VIS (Fischer Scientific, Leicestershire, UK) at a 490-nm wavelength. The total phenolic acids quantities were expressed in mg/g caffeic acid equivalents (CAE) (caffeic acid; Sigma-Aldrich, St. Louis, MO, USA) [33].

### 2.3. High-Performance Liquid Chromatography (HPLC-DAD) Analysis

Quantification of bioactive compounds from the microgreens of *Lepidium sativum* L. was performed using Agilent 1260 Infinity high-performance liquid chromatography (Agilent Technologies, Waldbronn, Germany) with a quaternary solvent manager coupled with a diode array detector (G1315C), degasser (G1311B), sample (G1329B), and column (G1316A) manager. Harvested *Lepidium sativum* L. was used to analyze the phenolic compounds, and during the analysis, the DAD collected data in the UV range of 210–400, in steps of 1 nm; while we used the following quantification wavelengths for individual analytes: 265 nm: 4-OH benzoic acid; 320 nm: caffeic acid; trans p-coumaric acid, ferulic acid, resveratrol, cinnamic acid; and 372 nm: myricetin, quercetin and kaempferol. One gram of the microgreen sample was dissolved in methanol (HPLC grade; 80%; Sigma-Aldrich, St. Louis, MO, USA), and the mixture was horizontally shaken (Unimax 2010; Heidolph Instrument, GmbH, Germany) at 25 °C, and 250 rpm for 8 h. Afterwards, the sample was filtered (84 g/m^2^ filter paper, Munktell, Germany) and extracted in 10 mL of 80% (*v*/*v*) methanol with a horizontal shaker. Before use, the standard solutions and the extract were filtered through a Q-Max syringe filter (0.22 mm, 25 mm; Frisenette ApS, Knebel, Denmark). For HPLC measurements we used selected standards in HPLC grade (4-OH benzoic acid, caffeic acid, trans p-coumaric acid, rutin, ferulic acid, myricetin, resveratrol, quercetin, cinnamic acid, kaempferol) purchased from Sigma-Aldrich, St. Louis, MO, USA, acetonitrile (HPLC grade; Sigma-Aldrich, St. Louis, MO, USA), phosphoric acid (ACS grade; Sigma-Aldrich, St. Louis, MO, USA), and double-deionized water (ddH_2_O) prepared with a purification system (Simplicity 185; Millipore SAS, Molsheim, France). HPLC measurements were performed on a Purosphere reverse phase C18 column (Merck, Darmstadt, Germany). The mobile phase D (acetonitrile) and the mobile phase C (0.1% phosphoric acid in ddH_2_O) were included in the mobile phase. The gradient elution was as follows: 0-1 min isocratic elution (90% C and 10% D), 1–6 min linear gradient elution (85% C and 15% D), 6–12 min (80% C and 20% D), 12–20 min (30% C and 70% D), and 20–25 min (30% C and 70% D). The initial flow rate was 1 mL/min, and the injection volume was 5 µL. The column thermostat was set as 30 °C, and the samples were kept at 4 °C in the sample manager. All obtained data were collected and processed using the Agilent OpenLab ChemStation software for LC 3D Systems [25].

### 2.4. Total Antioxidant Capacity

The free radical-scavenging potential of the experimental extract was evaluated using the 2,2-diphenyl-1-picrylhydrazyl (DPPH) method described by [34]. A 400 µL quantity of *Lepidium sativum* L. was mixed with 3,6 mL of DPPH working solution. This solution consisted of 0.025 g DPPH (Sigma-Aldrich, St. Louis, MO, USA) dissolved in 100 mL methanol (CentralChem, Bratislava, Slovakia). The absorbance of the mixture was measured using a spectrophotometer Jenway 6405 UV/VIS (Fischer Scientific, Leicestershire, UK) at a 515-nm wavelength. The free radical-scavenging potential was expressed in mg/g Trolox equivalents (TEAC) (Trolox; 6-hydroxy-2,5,7,8-tetramethylchroman-2-carboxylic acid; Sigma-Aldrich, St. Louis, MO, USA).

For the ABTS (2’-azinobis-(3-ethylbenzothiazoline-6-sulfonic acid; Merck, Darmstadt, Germany) radical scavenging assay, the procedure followed a method described previously [35] with a slight modification. A solution of cation-radical ABTS•+ was prepared by the reaction between 7 mM ABTS solution and 2.4 mM potassium persulfate solution (K2S2O8; Sigma-Aldrich, St. Louis, MO, USA). The final working solution of ABTS•+ was obtained after a reaction time of 14 h to 16 h at laboratory temperature in the dark. This reagent was diluted with acetate buffer (0.1 mol/L; pH 4.3), to achieve the absorbance of 0.700 units at 734 nm wavelength using a spectrophotometer (Shimadzu UV-1800; Cole-Parmer, IL, USA). After the addition of 50 µl of *Lepidium sativum* L. extract to 2950 µL of diluted ABTS•+ solution, the absorbance was measured at 20 min, after the initial mixing. Trolox was used as a standard substance. The results were calculated as mg/g Trolox equivalents (TEAC) of the sample, based on the calibration curve.

### 2.5. Leydig TM3 Cell Culture and Experimental Setups In Vitro

Murine Leydig cells were purchased from the American Type Culture Collection (ATCC #CRL-1714TM; Manassas, VA, USA). TM3 cell line was cultured in Dulbecco’s Modified Eagle’s Medium/Nutrient Mixture (Ham’s) F12, with HEPEs and phenol red for cell culturing/without phenol red for other experiments (DMEM/F12; Sigma-Aldrich, St. Louis, MO, USA), supplemented with 5% horse serum (HS; Gibco-Life Technologies, Auckland, New Zealand), 2.5 mM L-glutamine (Sigma-Aldrich, St. Louis, MO, USA), 2.5% heat-inactivated fetal bovine serum (FBS; BiochromAG, Berlin, Germany), and 1% penicillin/streptomycin solution (Sigma-Aldrich, St. Louis, MO, USA) at 37 °C in 5% CO_2_ and 95% saturated atmospheric humidity. TM3 cells were passaged after reaching 85–90% confluence and sub-cultured at 1:50 ratio in 75 cm^2^ flasks (TPP, Trasadingen, Switzerland). Growing cell culture was routinely controlled for microbial contamination and regular cell morphology. TM3 cells from passage no. 9 up to passage no. 27 were used for these experiments. Crude extract of *Lepidum sativum* L. was dissolved in DMSO. The vehicle concentrations did not exceed 0.5% (*v*/*v*). Vehicle-treated cells served as a negative control (NC) in each experiment. The 96- and 6-microwell plates were pre-coated overnight with gelatin (0.1% *w*/*v* in physiologically buffered saline) before cell seeding. Additionally, all obtained data were expressed as a percentage of the control (non-treated) group and experimental (treated) cells. At least, three separate repeats were carried out in each group.

### 2.6. Cytotoxic Assays

#### 2.6.1. Cell Viability

The viability of TM3 Leydig cells was evaluated using alamarBlue reagent (ThermoFisher Scientific, Invitrogen, Vantaa, Finland), as reported previously [27,35,36]. AlamarBlue assay is a sensitive oxidation-reduction indicator that fluoresces after the reduction of the blue color of resazurin to the pink color of resorufin, due to mitochondrial dehydrogenase activity in living cells [37]. In brief, Leydig cells were pre-cultured at an adjusted density (4 × 103 cells/well) in gelatin pre-coated 96-microwell plates, 24 h before the exposure. Afterward, the cell culture medium was removed, and fresh medium containing experimental doses (62.5–2000 µg/mL) of *Lepidium sativum* L. was applied for 24 h and 48 h. After the respective treatments, cells washed with Dulbeccos’s phosphate-buffered saline (DPBS; Sigma-Aldrich, St. Louis, MO, USA) were incubated with DMEM/F12 media (without phenol red) containing alamarBlue reagent in a final concentration of 5% (*v*/*v*). After 0.5 h min incubation in a CO_2_ incubator (37 °C; 5% CO_2_; and 95% atmospheric humidity), the fluorescence (excitation/emission: 530/590 nm wavelengths) was measured using a combined spectro-fluoro-luminometer GlomaxMulti+ (Promega Corporation, Madison, WI, USA).

#### 2.6.2. Cell Membrane Integrity

5-carboxyfluorescein diacetate, acetoxymethyl ester (CFDA-AM; Thermo Fisher Scientific, Waltham, Massachusetts, USA) is a nontoxic esterase substrate that can be metabolized by nonspecific esterase in living cells, from membrane-permeable and nonfluorescent molecules to polar, fluorescent carboxyfluorescein. This conversion indicates alterations in cell membrane integrity [38]. Briefly, TM3 cells were seeded at a density of 4 × 103 cells/well in gelatin pre-coated 96-microwell plates 24 h before the exposure. Pre-cultured Leydig cells were exposed to corresponding concentrations of *Lepidium sativum* L., starting from 62.5 to 2000 µg/mL for 24 h and 48 h. Subsequently, the medium was removed, and Leydig cells were washed with DPBS and incubated with fresh DMEM/F12 medium (without phenol red) containing CFDA-AM at a final concentration of 4 µM. After 0.5 h in a CO_2_ incubator (37 °C; 5% CO_2_; and 95% atmospheric humidity), the fluorescence (excitation/emission: 485/530 nm wavelengths) was measured with a combined spectrofoluoroluminometer GlomaxMulti+.

#### 2.6.3. Lysosomal Activity

A neutral red uptake (NRU; Thermo Fisher Scientific, Waltham, MA, USA) assay was used to evaluate the ability of viable cells to incorporate the supravital dye neutral red into cell membranes and concentrate in the lysosomes, where it binds to anionic and phosphate groups of the lysosomal matrix [39]. In brief, the murine Leydig cell line was grown at a density of 4 × 103 cells/well in gelatin pre-coated 96-microwell plates, 24 h before the exposure. Afterward, the cell culture medium was aspirated and TM3 cells were treated with experimental doses of *Lepidium sativum* L. (62.5 to 2000 µg/mL) for 24 h and 48 h. TM3 cells were washed with DPBS and incubated with NR at a final concentration of 0.005% (*w*/*v*) dissolved in DMEM/F12 (without phenol red) for 2 h in a CO_2_ incubator. The cells were washed repeatedly, and the dye was lysed with a buffer containing 1% (*v*/*v*) acetic acid in 50% (*v*/*v*) ethanol for 20 min. NR absorbance was measured at 525/660–720 nm wavelength, using a combined spectro-fluoro-luminometer GlomaxMulti+.

### 2.7. ROS Production

A nitroblue-tetrazolium (NBT; Sigma-Aldrich, St. Louis, MO, USA) assay was used for detection of the intracellular superoxide radical level. This is a colorimetric method conducted by assessing blue formazan deposits, formed by the reduction of the membrane-permeable and yellow-colored nitroblue-tetrazolium chloride (2,2’bis(4-nitro-phenyl)-5,5’-diphenyl-3,3’-dimethoxy-4,4’-diphenylene) diterazolium chloride; Sigma-Aldrich, St. Louis, MO, USA) by the superoxide radicals [40]. Mouse TM3 cells were pre-cultured at a density of 4 × 103 cells/well in 96-well plates for 24 h. Afterward, cell culture media was replaced by DMEM/F12 supplemented with corresponding doses (from 62.5 to 2000 µg/mL) of *Lepidium sativum* L. for 24 h and 48 h. Subsequently, the NBT salt was dissolved in DMEM/F12 containing 1.5% DMSO (final concentrations: 1mg/mL) and added to the Leydig cells. After a 3 h incubation (37 °C; 5% CO_2_ and 95% atmospheric humidity), the formed blue deposits were solubilized with 2M potassium hydroxide (KOH; p.a. CentralChem, Bratislava, Slovakia) dissolved in DMSO. The optical density was measured at a wavelength of 620 nm against 570 nm as a reference, using a microplate reader Multiscan FC (Thermo Fisher Scientific, Waltham, MA, USA).

### 2.8. Steroid Hormone Secretion

The production of testosterone was determined using an enzyme-linked immunosorbent (ELISA) assay. The ELISA method is an immunological reaction that combines the specific reactivity of antigens and antibodies with the efficient catalytic action of enzymes on substrates. TM3 cells were plated into 96-well plates at a density of 4 × 103 cells/well for 24 h. Pre-cultured cells were subsequently exposed to 62.5–2000 µg/mL of *Lepidium sativum* L. for 24 h and 48 h. After this time period, the cell culture media was aspirated from each well, centrifuged (3000 rpm; 10 min; 4 °C), and supernatants were stored in Eppendorf tubes at −80 °C until steroid determination. The procedure for progesterone and testosterone analyses was carried out according to the manufacturer’s instructions in ELISA kits (progesterone Cat. #K00225, testosterone Cat. #K00234, Dialab, Wiener Neudorf, Austria). The optical density was measured at a 450-nm wavelength using a microplate reader Multiscan FC. The sensitivities of both steroid hormones are presented in Table 1.

### 2.9. Gap Junctional Intracellular Communication Assay

The scalpel loading/dye transfer (SL/DT) assay relies on the introduction of small (MW < 900) nonpermeable dyes of lucifer yellow CH dilithium salt (LY; Sigma-Aldrich, St. Louis, MO, USA) into living cells, which are traced in their intercellular movement through the gap junctions. LY is negatively charged and it has a high fluorescence efficiency [41]. Leydig TM3 cells were seeded into 6-well plates at a density of 1.25 × 10^5^ cells/well for 24 h. After adhesion, cells were treated with experimental doses of *Lepidium sativum* L., starting from 62.5 to 2000 µg/mL for 48 h. After the respective treatments, a gap junction permeable tracer LY (final concentrations: 1mg/mL in calcium- and magnesium-supplemented PBS (CaMg-PBS; pH 7.2) was added to the cells and introduced into them with three parallel cuts made by a scalpel. After 6 min of incubation in the dark, the cells were washed with CaMg-PBS and fixed with 4% paraformaldehyde (PFA; Boster, Pleasanton, CA, USA). Finally, the images were captured using the Leica Application Suite X (LAS X) software, which is suitable for the fluorescent microscope DMI 6000 B (Leica Microsystems, Wetzlar, Germany), and a DCF 345FX camera. The distance at which the LY diffuses through the gap junction of TM3 cells was evaluated using ImageJ software 1.51 (National Institute of Health, LOCI, University of Wisconsin, USA) [42].

### 2.10. Statistical Analyses

All statistical analyses were performed using GraphPad Prism 6.01 (GraphPad Software Incorporated, San Diego, CA, USA). Descriptive characteristics (minimum, maximum, mean, and standard error of the mean, etc.) were evaluated at first. One-way analysis of variance (ANOVA) and Dunnett’s multiple comparison tests were used to examine differences between the *Lepidium sativum* L. treatments and the control groups. The geometric mean of the values from at least three (n = 3) independently repeated experiments was calculated and reported with a 95% confidence interval. The results were expressed as the as mean ± standard error of the mean (SEM).

## 3. Results

### 3.1. Biochemical Profile of Lepidium sativum L.

The accumulated data obtained from the biochemical assessment of *Lepidium sativum* L. are presented in Table 2. The total polyphenols, flavonoids, and phenolic acids in the experimental extract of microgreen were estimated, as follows. According to the Folin-Ciocalteu method, the total polyphenols content was 94.10 ± 6.72 mg GAE/g dry weight (d.w.). Further analyses of total flavonoid content confirmed 139.05 ± 7.19 mg QE/g d.w. In the case of total phenolic acids content evaluation, 70.89 ± 3.10 mg CAE/g d.w. was recorded.

### 3.2. Biologically Active Compounds and Antioxidant Capacity of Lepidium sativum L.

**Figure 1:**
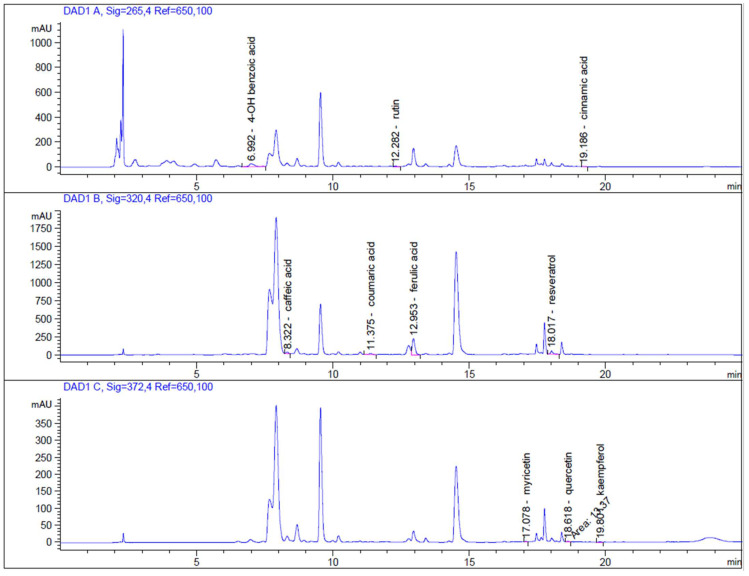
HPLC-DAD chromatograms of *Lepidium sativum* L. ethanolic extract measured at 265-nm (top chromatogram), 320-nm (middle chromatogram), and 372-nm (bottom chromatogram) wavelengths.

The accurate HPLC-DAD analysis was used as a suitable method for the identification and quantification of polyphenols in *Lepidium sativum* L. extract (Figure 1). Identification of monitored analytes was carried out by comparing retention times and UV spectra. From the analysis of phenolic compounds, ferulic acid (333.66 ± 0.64 mg/kg) and 4-OH benzoic acid (74.64 ± 0.62 mg/kg) were the most abundant molecules in the *Lepidium sativum* L. microgreen extract. Moreover, caffeic acid (28.69 ± 0.18 mg/kg), trans p-coumaric acid (22.67 ± 0.66 mg/kg), and cinnamic acid (1.56 ± 0.34 mg/kg) were also detected. Resveratrol (43.04 ± 0.11 mg/kg) was highly represented, followed by flavonoid glycosides rutin (23.31 ± 2.08 mg/kg) and quercetin (3.32 ± 0.06 mg/kg). Moreover, two flavonoid aglycones, myricetin (2.59 ± 0.10 mg/kg) and kaempferol (2.38 ± 0.03 mg/kg), were found in the extract. Except for these molecules, the HPLC-DAD detection method revealed a significant amount of chlorogenic acids. In particular, cryptochlorogenic acid (3552.83 ± 3.96 mg/kg) was the most abundant. The detected concentrations of biologically active molecules are summarized in Table 3. In addition, the DPPH method revealed that the free-radical scavenging activity of the *Lepidium sativum* L. microgreen was 9.43 (±0.01) mg TEAC/g d.w. Similarly, the ABTS method evaluated the scavenging potential, showing 105.95 (±0.01) mg TEAC/g d.w. Both parameters are summarized in Table 4.

### 3.3. Assessment of the Cytotoxic Effect of Lepidium sativum L.

#### 3.3.1. TM3 Metabolic Activity

**Figure 2:**
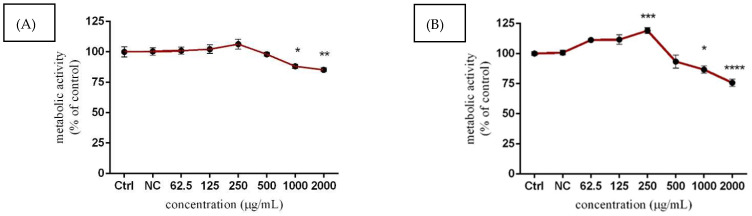
The effects of *Lepidium sativum* L. on TM3 cells metabolic activity after 24 h (**A**) and 48 h (**B**) exposure in vitro. Abbreviations: Ctrl—control group, NC—negative control. The data are presented as means (±SEM) optical density percent of the control (untreated) and experimental extract-treated groups. The data were collected from three independent experiments performed in triplicate. Levels of significance were established at **** (*p* < 0.0001); *** (*p* < 0.001); ** (*p* < 0.01), and * (*p* < 0.05). Statistical differences between the values of control and treated groups are indicated by an asterisk (*).

The analyses of the murine Leydig cells viability estimated using alamarBlue reagent revealed time- and dose-dependent effects. Higher concentrations of *Lepidium sativum* L. (1000 and 2000 µg/mL) displayed elevated cytotoxicity, since the metabolic activity of cells was significantly (*p* < 0.05; *p* < 0.01) decreased (88.21 ± 1.65% vs. 85.27 ± 1.60%) compared to the control (untreated) cells (100.00 ± 4.10%) after 24 h exposure (Figure 2A). This trend was more prominent (*p* < 0.0001) after 48 h treatment, whereas 2000 µg/mL resulted in a approx. 25% decrease in value, while 1000 µg/mL of *Lepidium sativum* L. also significantly (*p* < 0.05) reduced this parameter. Regarding the effects of lower tested concentrations, a significant (*p* < 0.01) elevation of metabolic activity was observed after a prolonged period of cultivation in samples corresponding to 250 µg/mL concentration (119.10 ± 2.44%), compared to the control group (100.00 ± 1.53%) (Figure 2B).

#### 3.3.2. TM3 Cell Membrane Integrity

**Figure 3:**
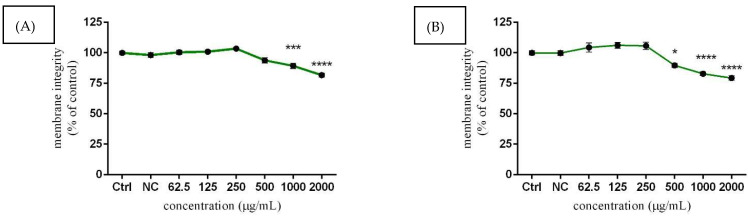
The effects of *Lepidium sativum* L. on TM3 cell membrane integrity after 24 h (**A**) and 48 h (**B**) exposure in vitro. Abbreviations: Ctrl—control group, NC—negative control. The data are presented as mean (±SEM) optical density percentage of the control (untreated) and experimental extract-treated groups. The data were collected from three independent experiments performed in triplicate. Levels of significance were established at **** (*p* < 0.0001); *** (*p* < 0.001), and * (*p* < 0.05). Statistical differences between the values of the control and treated groups are indicated by an asterisk (*).

Similar results to the metabolic activity assay were obtained in cell membrane integrity evaluation using the CFDA-AM method (Figure 3A) where the highest concentrations (1000 and 2000 µg/mL) of *Lepidium sativum* L. led to a significant (*p* < 0.001; *p* < 0.0001) decrease of cell membrane integrity (89.28 ± 2.14%; 81.68 ± 1.71%) after 24 h exposure compared to the untreated (control) cells (100.00 ± 1.48%). Similarly, significant (*p* < 0.0001) effects were more noticeable after prolonged exposures (82.91 ± 1.39; 79.34 ± 1.82%). In addition, the experimental concentration of 500 µg/mL caused increased cytotoxic effects, accompanied by membrane damages (89.78 ± 1.77%), with the treatment compared to control (100.00 ± 1.60%) (Figure 3B). On the other hand, lower concentrations of experimental extract did not negatively affect the cell membrane integrity, and the obtained data remained slightly above the control values.

#### 3.3.3. Lysosomal Activity of TM3 Cells

**Figure 4:**
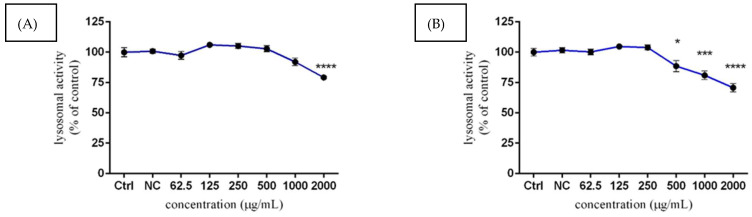
The effects of *Lepidium sativum* L. on TM3 cell lysosomal activity after 24 h (**A**) and 48 h (**B**) exposure in vitro. Abbreviations: Ctrl—control group, NC—negative control. The data are presented as mean (±SEM) optical density percentage of the control (untreated) and experimental extract-treated groups. The data were collected from three independent experiments performed in triplicate. Levels of significance were established at **** (*p* < 0.0001); *** (*p* < 0.001); and * (*p* < 0.05). Statistical differences between the values of the control and treated groups are indicated by an asterisk (*).

Neutral red uptake quantification was performed to identify related disruption of lysosomal activity and therefore possible cytotoxic effects of *Lepidium sativum* L. extract on TM3 murine Leydig cells. A significant (*p* < 0.001) reduction in lysosomal activity was observed only at 2000 µg/mL of experimental extract (79.27 ± 1.03%) after 24 h exposure, compared to the control group (100.00 ± 3.67%) (Figure 4A). However, loss of lysosomal activity, especially for the 1000 and 2000 µg/mL concentrations (81.02 ± 3.53% and 70.82 ± 3.43%), became more evident (*p* < 0.001; *p* < 0.0001) after 48 h treatment, compared to the untreated cells (100.00 ± 3.12) (Figure 4B). In the case of lower experimental doses of *Lepidium sativum* L., there were no observed significant changes after 24 h, or even after 48 h.

### 3.4. Assessment of ROS Production

**Figure 5:**
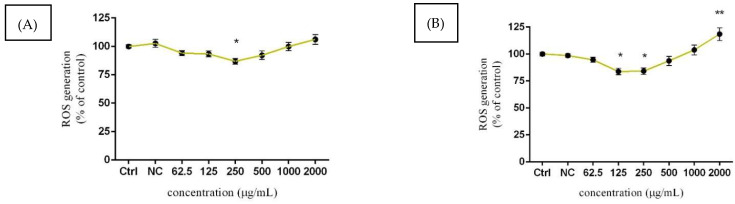
The effects of *Lepidium sativum* L. on ROS production after 24 h (**A**) and 48 h (**B**) exposure in vitro. Abbreviations: Ctrl—control group, NC—negative control. The data are presented as the mean (±SEM) optical density percentage of the control (untreated) and experimental extract-treated groups. The data were collected from three independent experiments, performed in triplicate. Levels of significance were established at ** (*p* < 0.01) and * (*p* < 0.05). Statistical differences between the values of the control and treated groups are indicated by an asterisk (*).

The intracellular formation of superoxide radical was evaluated using a nitroblue-tetrazolium (NBT) assay after 24 and 48 h exposure. After 24 h exposure (Figure 5A), the lower concentrations of the tested extract resulted in significant (*p* < 0.05) inhibition of superoxide radical production, where the value decreased by approx. 14% in comparison to the control (100.00 ± 1.52%). Similar significant (*p* < 0.05) tendencies were recorded at 125 µg/mL (83.83 ± 3.01%) and 250 µg/mL (84.15 ± 3.02%) after 48 h treatment (Figure 5B). In addition, a notable elevation regarding the superoxide radical production was observed in samples treated with the highest concentration (106.20 ± 4.34%). In the case of a prolonged period of exposure, the highest applied dose 2000 µg/mL of *Lepidium sativum* L. significantly (*p* < 0.01) increased the presented parameter compared to the untreated (control) cells (100.00 ± 5.98%).

### 3.5. Assessment of Steroid Hormone Secretion

**Figure 6:**
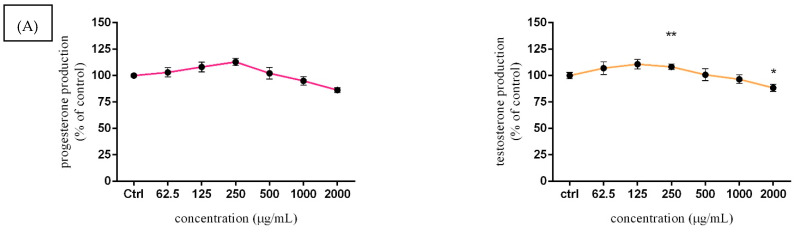

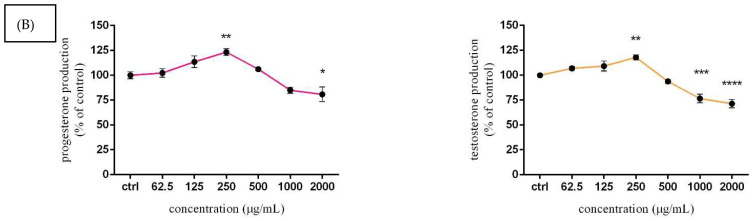
The effects of *Lepidium sativum* L. on TM3 cell production of sex-steroid hormones, after 24 h (**A**) and 48 h (**B**) exposure in vitro. Abbreviations: Ctrl—control group, NC—negative control. The data are presented as the mean (±SEM) optical density percentage of the control (untreated) and experimental extract-treated groups. The data were collected from three independent experiments performed in triplicate. Levels of significance were established at **** (*p* < 0.0001); *** (*p* < 0.001); ** (*p* < 0.01), and * (*p* < 0.05). Statistical differences between the values of the control and treated groups are indicated by an asterisk (*).

Taking into account both treatment periods (24 and 48 h), a bell-shaped dose–response curve was observed, as seen in Figure 6. A slight increase in progesterone and testosterone secretion, without significant changes was confirmed after 24 h exposure to *Lepidium sativum* L. Lower applied doses stimulated hormone secretion, observably at 125 µg/mL (108.20 ± 4.68%; 110.83 ± 4.46%) and 250 µg/mL (112.91 ± 3.34%; 108.40 ± 2.67%) compared to the control group (100.00 ± 1.34% vs. 3.01%). At 48 h exposure, 250 μg/mL of experimental extract caused a significant (*p* < 0.01) increase in progesterone (123.40 ± 3.36%) and testosterone (117.91 ± 2.61%) secretion. A higher applied dose of 2000 μg/mL (in progesterone) together with 1000 or 200 μg/mL (in testosterone) initiated a significant (*p* < 0.05; *p* < 0.001) decline compared to the control cells (100.00 ± 3.41% and 0.33%).

### 3.6. Assessment of GJIC Activity

**Figure 7:**
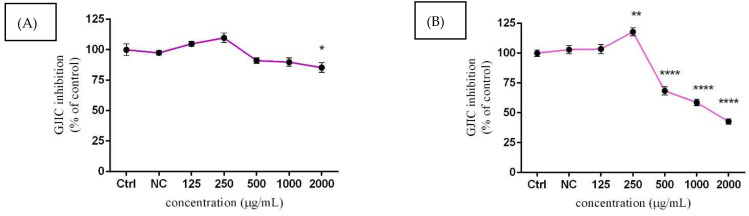
The effects of *Lepidium sativum* L. on GJIC inhibition after 24 h (**A**) and 48 h (**B**) exposure in vitro. Abbreviations: Ctrl—control group, NC—negative control. The data are presented as the mean (±SEM) optical density percentage of the control (untreated) and experimental extract-treated groups. The data were collected from three independent experiments performed in triplicate. Levels of significance were established at **** (*p* < 0.0001); ** (*p* < 0.01), and * (*p* < 0.05). Statistical differences between the values of control and treated groups are indicated by an asterisk (*).

**Figure 8:**
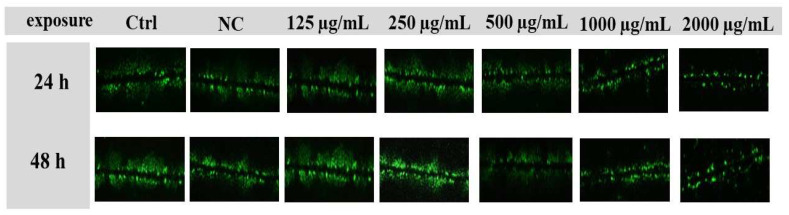
Representative images after 24 h and 48 h exposures to *Lepidium sativum* L., taken using the SL/DT technique. The LY dye spreading into the TM3 Leydig cells is related to the GJIC extent.

For the assessment of intercellular communication through gap junctions (GJIC) in TM3 cells, the SL/DT method was chosen. Moreover, the lowest experimental concentration (62.5 µg/mL) of *Lepidium sativum* L. was excluded from this evaluation, due to its mild effect, as confirmed by previous assessments. As displayed in Figure 7A, GJIC was positively affected at 125 µg/mL and 250 µg/mL, followed by gradual attenuation at higher doses after 24 h exposure. A significant (*p* < 0.05) inhibition of cellular communication was recorded at 2000 µg/mL (85.35 ± 3.95%) compared to the control group (100.00 ± 4.87%). Substantial changes in the presented parameter were observed after a prolonged time of cultivation. Significant (*p* < 0.01) stimulation of intracellular communication was confirmed with 250 µg/mL (117.91 ± 5.71%) of experimental extract. Furthermore, suppression of connexin channels activity was statistically proven (*p* < 0.0001) at 500 µg/mL (68.44 ± 5.86%), 1000 µg/mL (58.74 ± 4.71%), and 2000 µg/mL (42.71 ± 3.99%) of *Lepidium sativum* L. compared to the untreated cells (100.00 ± 4.61%) (Figure 7B). Representative images of GJIC activity are displayed in Figure 8.

## 4. Discussion

With accelerated economic development, lifestyle changes, and radical growth of environmental pollution, the incidence of male reproductive disruptions and subfertility has gained increased worldwide attention. Generally, several exogenous factors affect our health, and sufficient doses of biologically active compounds such as flavones, anthocyanins, lignans, and chlorogenic acids may stimulate cellular and organ physiological functions. In addition, the right cellular functions and microenvironment are essential for the health of an individual. The first part of our study characterized the unique properties of *Lepidium sativum* L., regarding the quality and quantity of biologically active molecules. The obtained results identified ferulic acid and 4-OH benzoic acid as the most abundant molecules, followed by resveratrol, rutin, quercetin, etc. In general, all of these have many positive effects, and their diverse structure could be related to numerous properties, such as antioxidant, anti-cancer, or anti-inflammatory effects [43,44]. In addition, the total polyphenols content was estimated at 94.10 (±6.72) mg GAE/g d.w., while further analyses of the total flavonoid content confirmed 139.05 (±7.19) mg QE/g d.w. The total phenolic acid content was detected at approximately 71 mg CAE/g d.w. The content of phenolic compounds and antioxidant activity of *Lepidium sativum* L. microgreens was evaluated previously [45]. The authors declared a highest measured phenolic content of around 1.54 g GAE/kg d.w., while the flavonoid content was estimated to be 13.94 g RE (rutin equivalent)/kg d.w. In the case of antioxidant capacity, the microgreens *Lepidium sativum* L. exhibited 0.12 mol/kg d.w. Reference [46] quantified the total polyphenols amount of several microgreen species, including *Amaranthus*, *Brassica japonica*, *Portulaca oleracea*, and *Lepidium sativum*. The highest content was established in *Portulaca oleracea* (13581 µg/g d.w.), while *Lepidium sativum* had notably lower phenolic contents (4354 µg/g d.w.). In addition, the authors measured the antioxidant activity of experimental microgreens using the 2,20-azinobis 3-ethylbenzothiazoline-6-sulfonic acid (ABTS) method. The gained results demonstrated widely variable antioxidant activities. The highest antioxidant activity was observed in *Lepidium sativum,* at 92.60 mmol TEAC/100 g d.w., followed by *Amaranth* (65.70 mmol TEAC/100 g d.w.). In accordance with our results, we can conclude that *Lepidium sativum* L. reported significant levels of polyphenol, flavonoids, and phenolic acid, which could suggest health-promoting effects and a potential to modulate physiological functions and the cellular environment. It is necessary to emphasize that the ratio of individual polyphenols to the other types may vary distinctly during sprouting, and therefore differences in total phenol contents might be explained by the physiological state of *Lepidium* variants. Moreover, although it is generally stated that microgreens are characterized by high amounts of secondary metabolites compared to their mature counterparts [47,48], further studies revealed different, more nuanced conclusions.

Aside from analyses focused on the biochemical profile of *Lepidium sativum* L., HPLC-DAD identification of some specific polyphenols was conducted. As mentioned above, 4-OH benzoic acid, and ferulic acid, followed by resveratrol, rutin, quercetin, kaempferol, etc. were the most abundant. Besides this, the HPLC-DAD detection method revealed a significant amount of chlorogenic acids. In particular, cryptochlorogenic acid (3552.83 ± 3.96 mg/kg) was the most abundant. A similar proportion of biologically active compounds was confirmed by a previous study [46]. In accordance with our results, chlorogenic acid was the most abundant (998 µg/g d.w.), followed by feruloyl quinic acid (971.1 µg/g d.w) and rutin (1085 µg/g d.w). Elevated levels of p-coumaric acid, ferulic acid, kaempferol-7-O-glucoside, and caffeic acid were further observed. On the base of HPLC analysis, the authors of [49] determined major and minor biologically active molecules present in *Lepidium sativum* callus culture. Kaempferol (2.64 mg/g d.w.), quercetin (7.58 mg/g d.w), and chlorogenic acid (2.86 mg/g d.w) were categorized as major phytochemicals accumulated in this plant. Aside from these, ferulic acid, p-coumaric acid, and caffeic acid were detected at lower concentrations compared to our measurements. In addition, vanillic acid and sinapic acid were not detected in our case, while the results of the previous study confirmed minor levels of both.

It is evident that the microgreens of *Lepidium sativum* L. are a rich source of biologically active molecules, which could be useful in antioxidant defense and redox homeostasis. In addition, many recent studies have confirmed the stimulatory and protective effects on male reproductive functions. They are significantly able to protect cellular functions and processes such as spermatogenesis, sperm motility, viability, fertilization activity, as well as sex-steroid hormone production, libido activity, and prevention of oxidative stress onset [50,51,52,53]. Concerning this information, there is a need to determine the essential cellular parameters and molecular pathways, which could help to understand the mechanism of action. Potential cytotoxic effects of *Lepidium sativum* L. were investigated using various methods, including alamarBlue, CFDA-AM, and neutral red uptake (NRU) assays. According to our experimental data, *Lepidium* extract displayed mild cytotoxicity toward TM3 Leydig cells, since only the highest tested concentrations (1000 and 2000 µg/mL) decreased the membrane integrity and metabolic activity after 24 h and 48 h exposure. The cytotoxicity of *Lepidium sativum* L. was evaluated in a previous study [54]. Human liver cells HepG2 were exposed to different experimental concentrations (5–500 µg/mL) of *Lepidium* extract for 24 h. Cell viability, lysosomal activity, and changes in cell morphology were evaluated by mitochondrial cytotoxicity (MTT) assay or using an NRU assay. The results showed that lower applied doses (from 5 to 25 µg/mL) did not significantly affect the cell viability of the treated cells. However, the highest dose of extract (250 and 500 µg/mL) caused a significant decline in mitochondrial and lysosomal activity. In addition, both of these concentrations affected cell morphology, adhesion, proliferation, and cell growth. The cytotoxicity of *Lepidium sativum* hydro-alcoholic extract was also confirmed for K562 leukemia cells [55]. This cellular system was treated with 12.5, 25, 50, and 100 µg/mL of *Lepidium* at different time intervals (24, 48, and 72 h). The results revealed time- and dose-dependent cytotoxicity. The highest dose (100 µg/mL) progressively inhibited cell viability, as evaluated by the MTT assay, at all time periods. Moreover, the determination of morphological changes confirmed the fragmentation of chromatin and the transformation of the spherical shape of cells at 25 µg/mL. Compared to our results, the discussed studies confirmed the hypothesis about significant dose- and time-dependent cytotoxic effects. Additionally, it seems that cancer cell lines could display a higher sensitivity toward *Lepidium sativum* treatment. Matching results were obtained by [56], where cancerous lines (human colon cancer DLD-1 and endometrium cancer cell ECC-1) responded to the tested extract more evidently compared to peripheral lymphocyte cells (PML). Decreased lysosomal integrity was detected only in samples after a 1000 µg/mL treatment using both PML cells.

The oxidative stress induced by many current environmental pollutants has been implicated in the initiation of several pathological processes, which could negatively affect the overall health of humans and wildlife. The male reproductive system is particularly susceptible to oxidation damage, and the testicular microenvironment, as well as the germ, sperm, Sertoli, and Leydig cells, could be irreversibly disrupted. In addition, disruption of the common processes maintaining sufficient production of spermatozoa and sex-steroid hormone production could reduce the overall reproductive capacity. Results obtained from our experimental study suggest that lower concentrations of *Lepidium sativum* L. have significant antioxidant potential but increasing doses of *Lepidium* caused a progressive upturn in superoxide radical production. A recent study [57] showed, that oral administration of *Lepidium* affects testicular tissue and improves semen parameters. Twenty-four C57BL/6 male mice received 50 mg/kg of *Lepidium* orally for 30 days. Subsequently, morphometry, spermatozoa concentration, motility, as well as oxidative stress were evaluated. Experimental data revealed that the applied extract had a beneficial effect related to the decrease in oxidative stress, which could explain the improvement of the fertility of mice. Moreover, the authors confirmed a significant antioxidant effect correlated with progressive recovery in tetrahydrocannabinol-treated sperm damage. The protective effect of *Lepidium sativum* extract against oxidative stress has been determined previously [54]. Human liver cells HepG2 were treated at biologically-safe concentrations, starting at 5 to 25 µg/mL for 24 h, and then 0.25 mM of hydrogen peroxide was added. Intracellular ROS production was quantified using the 2,7-dichlorofluorescein diacetate (DCFH-DA) method, while lipid peroxidation was measured using the thiobarbituric acid-reactive substances (TBARS) protocol. According to the results, an applied dose of hydrogen peroxide significantly increased ROS generation, while pre-treatment with *Lepidium* at 5, 10, and 25 µg/mL significantly reduced the levels of ROS production. A similar tendency was recorded for lipid peroxidation. HepG2 cells pre-treated with the same concentrations of *Lepidium* showed significant inhibition in this parameter, compared to cells treated with hydrogen peroxide alone. Other markers of oxidative stress were evaluated in [58]. Albino rats were administered to *Lepidium sativum* L. (20 mg/kg), as well as to parallel exposure to *Lepidium* and AlCl_3_ (10 mg/kg) for 8 weeks. Exposure to AlCl_3_ resulted in increased ROS production in the liver and kidney, accompanied by a decline in glutathione (GSH) and catalase (CAT) activity. Moreover, ferric reducing antioxidant power (FRAP) was significantly reduced. Moreover, these inhibitions were statistically alleviated by *Lepidium* administration. Progressive differences in CAT and GSH levels were recorded in rats receiving *Lepidium* along with AlCl_3_, compared to those receiving *Lepidium* after that. In addition, *Lepidium* alone significantly increased the levels of antioxidant indicators, such as FRAP, CAT, and GSH. We may conclude that, although the microgreen *Lepidium sativum* may be considered an effective herb in various current disorder treatments, the male reproductive system is a sensitive indicator of physiological deviations, with an immediate onset of intra- and -intracellular changes in the testis compartment. It is essential to take into account multilevel processes, including steroidogenesis and spermatogenesis.

Since steroidogenesis is one of the key processes during the development of healthy sperm cells in an organism, we investigated the effect of *Lepidium sativum* L. on basal production of steroid hormones in TM3 Leydig cells, namely progesterone and testosterone. A significant stimulation was confirmed at 250 µg/mL of *Lepidium*, followed by a statistical decrease at 2000 µg/mL after 48 h exposure. A similar effect was recorded in the testosterone evaluation (48 h), while a significant stimulation was shown at 250 µg/mL after 24 h cultivation. A previous study [15] evaluated the potential of *Lepidium sativum* extract to modulate hormone levels in the blood of the male mice, as well as the possibility of increasing fertilizing ability. Albino Swiss mice were orally treated with 20 mg/mL of *Lepidium* for 2 weeks. Spermatozoa parameters, such as concentration, viability, and motility were supplemented by histological evaluations and hormonal level measurements. The results obtained revealed a significant stimulation of reproductive parameters when the motility and viability of mice spermatozoa were increased. In the case of testosterone production, significant growth was recorded in the group treated with *Lepidium sativum* compared to the control group of animals. The repro-stimulating activity of bioactive compounds present in *Lepidium sativum* was confirmed in [53]. NMRI mice were administered 200, 400, and 600 mg/kg body weight of *Lepidium* and coenzyme Q10 (200, 300, and 400 mg/kg body weight) for 2 weeks. Sexual behavior, spermatozoa parameters, and hormone secretion were evaluated. The experimental data indicated that the highest dose of *Lepidium* together with 200 mg/kg of coenzyme Q10 exhibited a radical stimulating effect on studied parameters. In particular, testosterone secretion was significantly increased after the respective treatment. In another study [16], rabbit blood was collected for hormonal status determination after administration of 5%, 7%, and 10% of Lepidium sativum powder after 8 weeks. The results showed that dietary inclusion of Lepidium significantly increased luteinizing hormone (LH) secretion at 7% and 10% doses. Moreover, there was no significant difference in the average plasma testosterone concentrations after 8 weeks of administration. Although the final effect of *Lepidium sativum* in the biological system may potentially vary and depend on several factors, such as the type of organism, duration, and method of extract administration, changes in the level of sex-steroid hormones could be linked to the inhibition of gap junctional intracellular communication.

The role of GJIC in maintaining many physiological functions has been sufficiently proven. It is crucial in modifying cell growth and cell death, with a specific role in the context of the male reproductive system. Direct interaction of testicular cells via intercellular junctions allows Sertoli, Leydig, and germ cells to provide an optimal microenvironment for proper functions and to support organization into well-structured tissue [59]. At the same time, testicular GJIC is the major molecular regulator of male fertility, and they can control reproductive functions in multiple steps, such as sex-steroid hormone production, spermatogenesis, and sperm maturation [60]. The results of our study confirmed dose-depended changes in GJIC activity in TM3 Leydig cells after *Lepidium sativum* L. treatment in vitro. A prolonged time of cultivation and lower experimental doses (250 µg/mL) caused a significant stimulation of this parameter, while the increasing concentration of *Lepidium* suggests a significant decline in this parameter. Currently, there are no scientific data that can explain the potential changes in GJIC activity after *Lepidium sativum* L. exposure. However, we may describe the effect of major biologically active compounds occurring in *Lepidium* on connexin 43 (Cx43) channels, primarily expressed in testicular tissue. The abilities of herbal extracts to modify GJIC activity were confirmed by our recent study [27]. The authors stated that higher experimental doses of Levisticum and Calendula, which are rich in flavonoids and chlorogenic acids, could significantly affect intercellular communication and consequently affect sex-steroid hormone secretion in murine Leydig cells. According to a previous study [61], quercetin has a significant ability to increase Cx43 expression in NMRI strain male mice. Quercetin was dissolved in distilled water at 75 and 150 mg/kg body weight and treated for 35 days. The results revealed, not only progressive stimulation of GJIC activity, but also an interesting potential of quercetin to initiate GJIC recovery after Pb exposure. Furthermore, the authors of [62] reported that resveratrol could increase Cx43 expression on the membrane of parental HCT116 and parental CT26 cell lines. Likewise, references [63,64] indicated that resveratrol supports the expression of Cx43 and GJIC activity in cancer cells, including hepatocellular carcinoma and melanoma.

## 5. Conclusions

The results of our in vitro study confirmed a rich biochemical profile with the exact identification of biologically active molecules, followed by a significant antioxidant capacity of *Lepidium sativum* L. The results also revealed that exposure to this microgreen has a time- and dose-dependent effect on the cellular parameters monitored in this study. The lower experimental doses did not show cytotoxic potential, and doses up to 250 µg/mL indicated an increasing stimulatory potential, especially in sex-steroid hormone secretion, accompanied by an increase in GJIC activity. Moreover, increased experimental doses initiated inhibition in cell viability, membrane integrity, and lysosomal activity. Moreover, these doses could increase ROS generation and through GJIC inhibition may cause sex-steroid hormone depletion. We may conclude that a sufficiently balanced administration of the microgreen *Lepidium sativum* L. could preventatively affect the cellular and molecular fundamentals of the male reproductive system.

## Figures and Tables

**Table 1:** Intra-assay, inter-assay variability, and sensitiveness for the selected steroid hormones.

Hormone	Intra-Assay Variability (%)	Inter-Assay Variability (%)	Sensitivity
**Progesterone**	≤4.0	≤9.3	0.05 ng/mL
**Testosterone**	≤7.0	≤8.3	0.10 ng/mL

**Table 2:** Biochemical profile of the *Lepidium sativum* L.

Parameter	Concentration
**The total polyphenols content**	94.10 ± 6.72 mg GAE/g d.w.
**The total flavonoids content**	139.05 ± 7.19 mg QE/g d.w.
**The total phenolic acids content**	70.89 ± 3.10 mg CAE/g d.w.

Abbreviations: Data are presented as means (±SEM) from three independent measurements. GAE—gallic acid equivalents, QE—quercetin equivalents, CAE—caffeic acid equivalents, d. w.—dry weight.

**Table 3:** Biologically active compounds of *Lepidium sativum* L. and its antioxidant capacity.

Phenolic Compounds	Concentration (mg/kg d.w.)
**4 -OH benzoic acid**	74.64 (± 0.62)
**caffeic acid**	28.69 (± 0.18)
**trans p-coumaric acid**	22.67 (± 0.66)
**rutin**	23.31 (± 2.08)
**ferulic acid**	333.66 (± 0.64)
**myricetin**	2.59 (± 0.10)
**resveratrol**	43.04 (± 0.11)
**quercetin**	3.32 (± 0.06)
**cinnamic acid**	1.56 (± 0.34)
**kaempferol**	2.38 (± 0.03)

Abbreviations: Data are presented as means (±SEM) from three independent measurements. d.w.—dry weight.

**Table 4:** Antioxidant capacity of *Lepidium sativum* L.

Parameter	Value
**DPPH assay**	9.43 ± 0.01 mg TEAC/g
**ABTS assay**	105.95 ± 0.01mg TEAC/g

Abbreviations: Data are presented as means (±SEM) from three independent measurements. d.w.—dry weight, TEAC—Trolox equivalents.

## Data Availability

The datasets generated in this study are available upon request to the corresponding author.
